# Role of Artificial intelligence model in prediction of low back pain using T2 weighted MRI of Lumbar spine

**DOI:** 10.12688/f1000research.154680.1

**Published:** 2024-09-10

**Authors:** Ali Muhaimil, Saikiran Pendem, Niranjana Sampathilla, Priya P S, Kaushik Nayak, Krishnaraj Chadaga, Anushree Goswami, Obhuli Chandran M, Abhijit Shirlal

**Affiliations:** 1 Department of Medical Imaging Technology, Manipal College of Health Professions, Manipal Academy of Higher Education, Karnataka, Manipal, 576104, India; 2 Department of Biomedical Engineering, Manipal Institute of Technology, Manipal Academy of Higher Education, Manipal, 576104, India; 3 Department of Radio Diagnosis and Imaging, Kasturba Medical College, Manipal Academy of Higher Education, Karnataka, Manipal, 576104, India; 4 Department of Computer Science and Engineering, Manipal Institute of Technology, Manipal Academy of Higher Education, Manipal, 576104, India

**Keywords:** Deep learning, Machine learning, low back pain, intervertebral discs, lumbar vertebrae

## Abstract

**Background:**

Low back pain (LBP), the primary cause of disability, is the most common musculoskeletal disorder globally and the primary cause of disability. Magnetic resonance imaging (MRI) studies are inconclusive and less sensitive for identifying and classifying patients with LBP. Hence, this study aimed to investigate the role of artificial intelligence (AI) models in the prediction of LBP using T2 weighted MRI image of the lumbar spine.

**Methods:**

This was a prospective case-control study. A total of 200 MRI patients (100 cases and controls each) referred for lumbar spine and whole spine screening were included. The scans were performed using 3.0 Tesla MRI (United Imaging Healthcare). T2 weighted images of the lumbar spine were segmented to extract radiomic features. Machine learning (ML) models, such as random forest, decision tree, logistic regression, K-nearest neighbors, adaboost, and deep learning methods (DL), such as ResNet and GoogleNet, were used, and performance measures were calculated.

**Results:**

Our study showed that Random forest and AdaBoost are the most reliable ML models for predicting LBP. Random forest showed high performance with area under curve (AUC) values from 0.83 to 0.88 across all lumbar vertebrae and L2-L3, L3-L4, and L4-L5 intervertebral discs (IVDs), with AUCs of 0.88 the highest at L5-S1 IVD (0.92). Adaboost demonstrated high performance at the L2-L5 vertebrae with AUC values of 0.82 to 0.90, with the highest AUC (0.97) at the L5-S1 IVD. Among the DL models, GoogleNet outperformed the other models at 30 epochs with an accuracy of 0.85, followed by ResNet 18 (30 epochs) with an accuracy of 0.84.

**Conclusion:**

The study demonstrated that ML and DL models can effectively predict LBP from MRI T2 weighted image of the lumbar spine. ML and DL models could also enhance the diagnostic accuracy of LBP, potentially leading to better patient management and outcomes.

## Introduction

Low back pain (LBP) is the most prevalent musculoskeletal condition worldwide and the leading cause of disability. In 2019, it held the 9 ^th^ position in disability-adjusted life years (DALYs) accounting for 2.5% of the overall “DALYS.” LBP was the primary cause of years lived with disability (YLDs), representing 7.41% of the total YLDS. In 2020, there were more than half a billion prevalent cases of LBP globally, and projections indicate that this number will exceed 800 million by 2050. Although age-standardized rates have slightly decreased over the past three decades, the number of LBP cases continues to increase owing to population growth and aging, particularly in Asia and Africa. ^1^^,^^2^ LBP can be caused by various factors, including lifestyle, psychological, and social factors. To reduce the incidence of LBP, it is essential to address modifiable risk factors, such as smoking and obesity, which are associated with a high risk of developing condition. ^3^^,^^4^ LBP can result from injuries or degenerative changes in the lumbar region, including facet joints, intervertebral discs (IVD’s), ligaments, and muscles. It is also associated with annual tears, disc height reduction, facet degeneration, and end-plate abnormalities such as Schmorl’s nodes, fractures, erosion, and calcifications. ^5^^–^^8^

MRI of the spine is a noninvasive technique that is regarded as the gold standard for detecting and diagnosing spinal diseases. T2 weighted MRI enhances tissue contrast and offers greater sensitivity than traditional CT imaging for diagnosing conditions such as IVD herniation, nerve root entrapment, and spinal canal stenosis. MRI can identify IVD degeneration and vertebral endplate changes, which are associated with clinically significant LBP. Imaging studies have revealed that 87% of asymptomatic individuals also exhibit lumbar IVD abnormalities on MRI. ^9^^–^^13^

Radiomics is a vital medical technique used in clinical practice for evaluation, diagnosis, selection of a course of treatment, and monitoring. Radiomics, a rapidly advancing artificial intelligence (AI) method in medical imaging, can objectively, reproducibly, and efficiently extract numerous quantitative features from medical images. These features are used to develop radiomic models or signatures that aid in interpreting various clinical phenotypes, such as patient genotyping, treatment efficacy, and clinical outcomes. ^14^^–^^17^

AI encompasses systems that can generate accurate interference from large datasets using advanced computational algorithms. Similar to humans, machines require learning for intelligent behavior. Therefore, AI includes various learning algorithms, such as machine learning (ML) and increasingly popular deep learning (DL) algorithms. Although AI originated in the 1950s, its development has accelerated since 2000, owing to advancements in computational power. Currently, AI technology provides indispensable tools for intelligent data analysis, particularly for solving medical diagnostic problems. The relationship between radiomics and AI is symbiotic. The high-dimensional nature of radiomics demands powerful analytic tools, and AI, with its advanced capabilities, is well-suited for this task. Conversely, AI applications with medical images rely on radiomics because the metrics used to train and build AI models are derived from radiomic approaches, specifically through feature extraction and feature engineering techniques. ^18^^–^^26^

Few studies have assessed the utility of radiomics-based ML models and DL techniques for predicting LBP. Hence, this study aimed to investigate the role of AI models in the prediction of LBP using T2 weighted MRI image of the lumbar spine.

## Methods

This was a prospective, case-control study. The institutional ethical committee (IEC2:179/2023) was obtained from Kasturba medical college and Hospital, Manipal, India on 20 ^th^ July 2023, followed by the Clinical trial registry (CTRI) registration: CTRI/2023/08/056954, 25/08/2023, https://ctri.nic.in/Clinicaltrials/login.php. Written informed consent (IC) was obtained from all the participants.

### Eligibility criteria

Patients referred for MRI of the lumbar spine and whole-spine screening were included. The patients were screened using the questionnaire “Delphi definitions of low back pain prevalence (DOLBaPP)” ^27^ questionnaire to check for LBP prevalence. Patients were considered symptomatic if they experienced LBP for 12 months. Patients were considered asymptomatic if they experienced no current back pain and no memory (severe or disabling) back pain. A total of 100 cases and controls (Mean age; cases: 48.48±16.1 years; controls: 51.46±18.4 years) were included. Demographic details of the patients are shown in Table 1. Patients with tumors, severe osteoporosis, or previous spine surgeries were excluded.

**Table 1.** Showing the demographic details of the population.

	Cases (Symptomatic)	Controls (Asymptomatic)
Subject (n)	100	100
Age in years (Mean±SD)	48.48±16.1	51.46±18.4
Gender	42 (Females)	40 (Females)
	58 (Males)	60 (Males)

**MRI Image acquisition:** All MRI scans were performed with 3.0 Tesla MRI (United Imaging uMR 780). The MRI image acquisition parameters are listed in Table 2.

**Table 2.** showing the acquisition parameters of T2 weighted MRI Lumbar spine.

Parameter	Sagittal T2
Sequence	Fast Recovery Fast Spin echo (FRFSE)
TR (msec)	2494
TE (msec)	100
Matrix size	224 × 199
Slice thickness (mm)	3.5
Flip angle (Degrees)	90

### Segmentation and radiomic feature extraction

**Figure 1.**
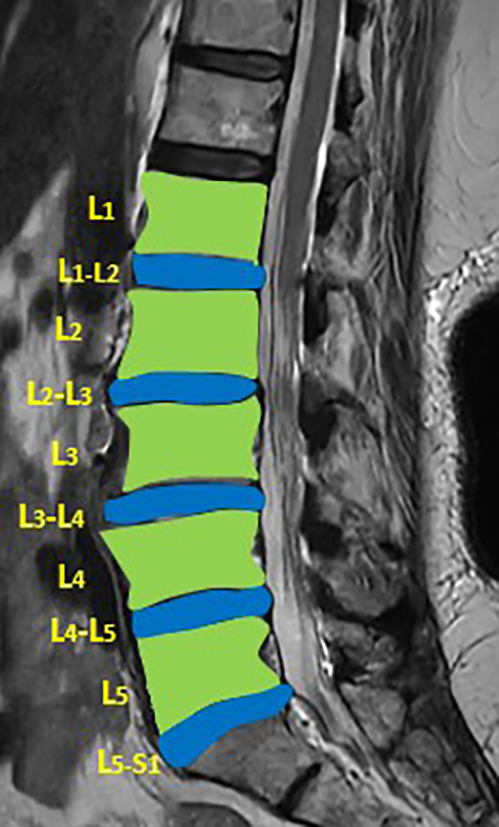
Showing the segmentation of lumbar vertebrae and intervertebral disc on T2 weighted image.

The DICOM MRI images of MRI T2 weighted images of the lumbar vertebrae and disc space for each patient were loaded into the 3D slicer software (Version 4.10.2). Segmentation of the lumbar vertebrae and intervertebral discs (IVD) was performed manually ( Figure 1). Radiomic features from the lumbar vertebrae and IVDs were extracted for both the cases and controls (Supplementary file 1).

### Machine learning model

ML classifiers such as Random Forest, Decision tree, logistic regression, k-nearest neighbors (KNN), and AdaBoost were used. The ML classifiers were run in the Conda virtual environment, which was integrated with Python (version 3.9.7). ^28^ Several libraries such as NumPy, ^29^ scikit, ^30^ pandas, ^31^ seaborn, ^32^ matplotlib, ^33^ and others were installed to support the analysis. The training of the models utilized 8 GB of RAM, along with an Intel ^®^core ^TM^ i5 Central Processing Unit (HP ProBook 440). The study was conducted on a 64-bit Windows operating system to run the classifiers.

**Data normalization:** This is an essential step because it assigns equal weight to each variable, preventing any single variable from disproportionately influencing the model performance owing to its large numerical values. In our study, the min-max normalization (rescaling) technique was employed for the entire dataset.

**Feature selection:** Mutual information method was used for the feature selection of the top 20 radiomic features at each lumbar vertebra and IVD for both cases and controls.

### Model training and validation

The data were split into training and testing ratios of 80:20. The data were subjected to five-fold cross-validation, where different subsets were trained to assess model efficiency. The input data were split into five equal parts: four groups for training and five for testing using various permutations and combinations in the cross-validation process. The parameters were hypertuned using a grid search technique that automates this tuning to determine the best values.

### Performance metrics of the ML models

The performance metrics of the ML models for the test dataset were assessed using accuracy, precision, F1 score, area under the curve (AUC), Hamming loss, Jaccord score, log loss, and Mathew’s correlation coefficient (MCC).

Validation of the testing model from the confusion matrix was assessed using

Accuracy=((TP+TN)/(TP+TN+FP+FN))

Precision=(TP/(TP+FP))=PPV

Recall=(TP/(TP+FN))

F1=(2_(Precision_Recall)/(Precision+Recall))

Where, TP - True positive, TN - True negative, FP - False positive, FN - False negative, PPV - Positive predictive value.

**Figure 2.**
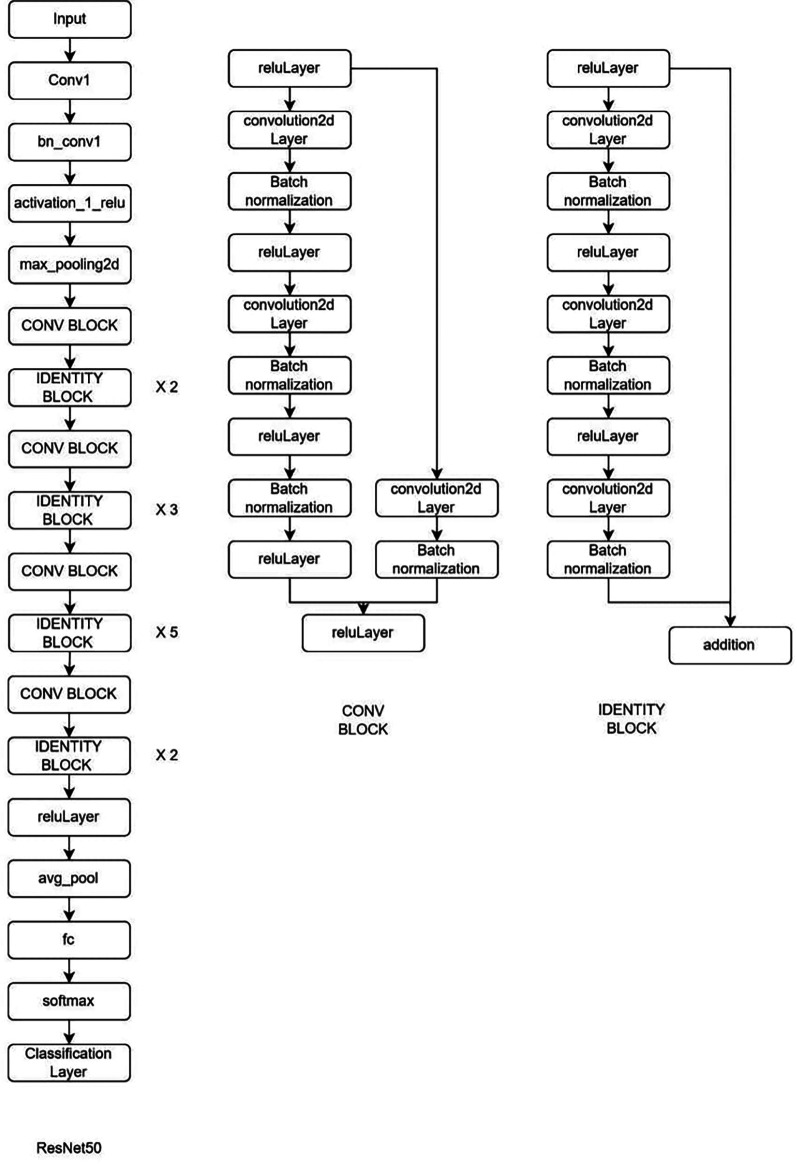
Architectural configuration delineating the structure of ResNet50.

**Figure 3.**
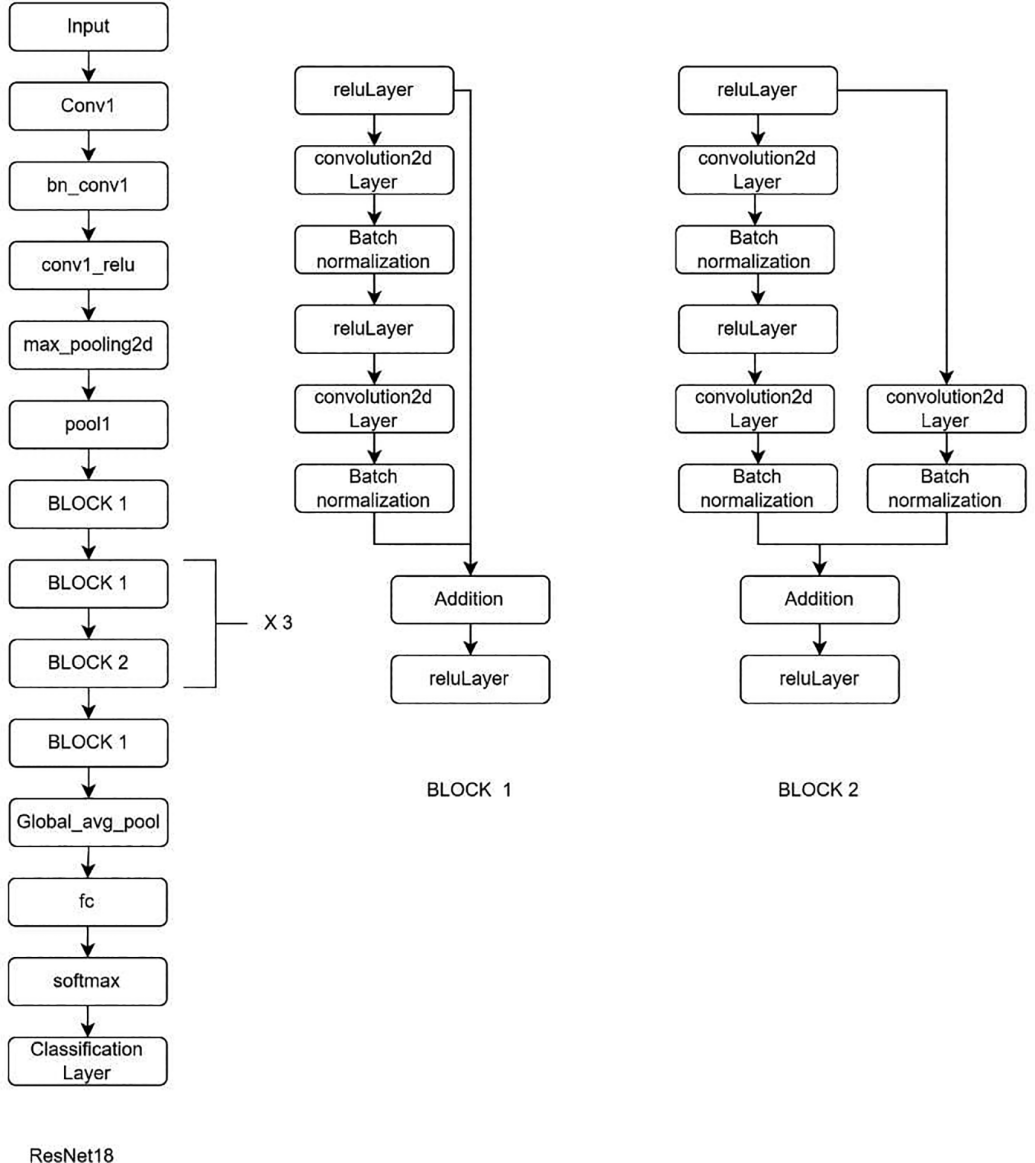
Architectural configuration delineating the structure of ResNet18.

**Figure 4.**
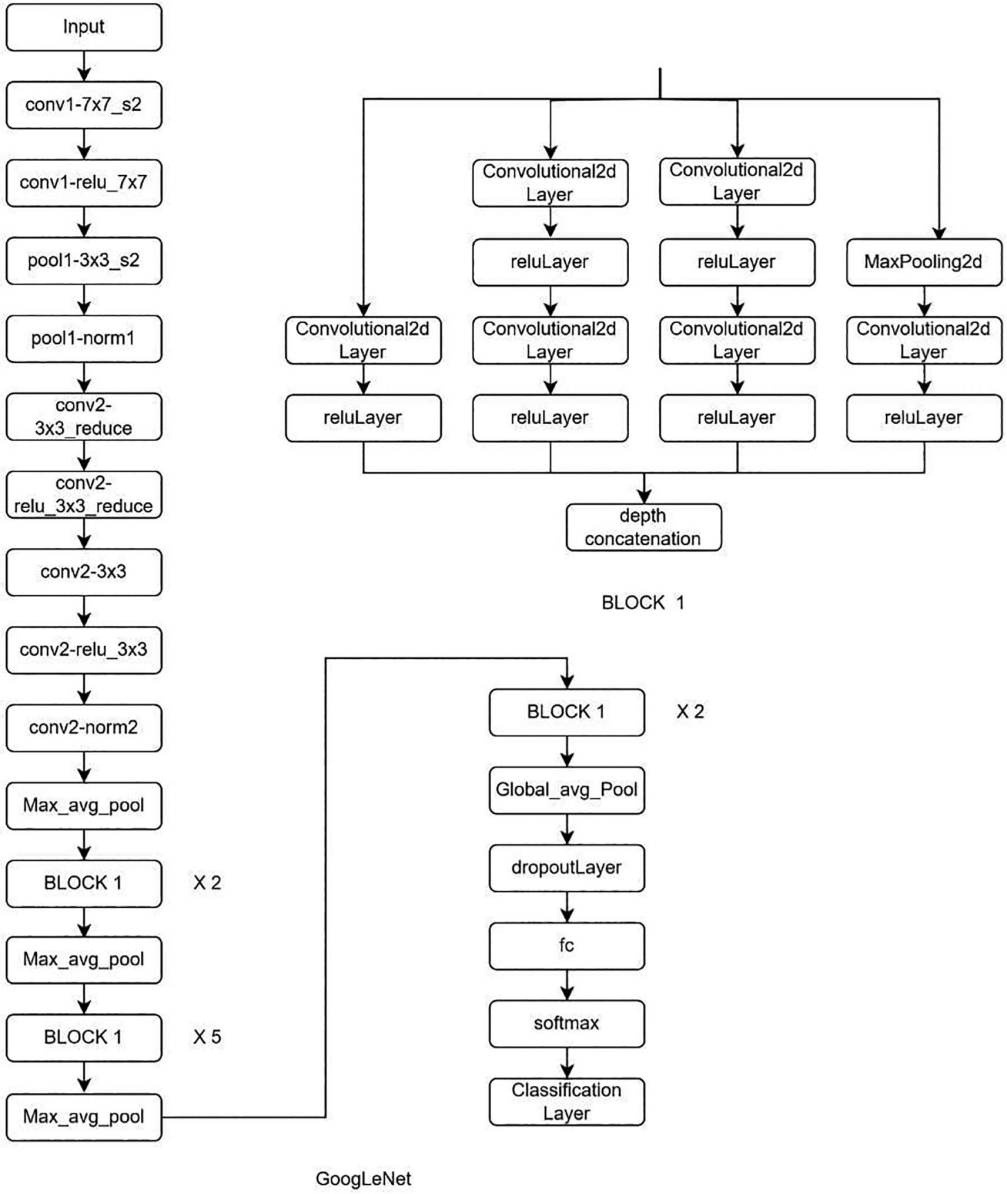
Architectural configuration delineating the structure of GoogleNet.

**Deep learning Model:** MRI Images of the lumbar spine were collected in the Joint Photographic Experts Group (JPEG) format. The input images were cropped and resized to 184 × 282 pixels to mainly include the lumbar vertebrae and disc space. Intensity normalization was performed on all images such that the pixel values across multiple images were normalized to the same statistical distribution, facilitating improved analysis of MRI images. Further as an assistance DL, a subset of AI, was used. Transfer learning is a DL technique in which pre-trained networks are utilized to train the model for custom usage. One of the major advantages of this method is that it avoids training the network from scratch by using weights that are trained on the 1000 class ImageNet dataset. There are multiple pre-trained models in which GoogleNet and ResNet (18 and 50) were used ( Figures 2– 4). These are convolutional neural networks, meaning that convolution layers play a major role. These are feature extraction layers that perform convolution with different kernels.

GoogLeNet is a part of the inception model, which is a 22-layer deep network that is computationally efficient. On the other hand, ResNet has an advantage over other networks because it consists of skip connections, which avoids the vanishing gradient.

The dataset was divided into a 90:10 training and test split ratio. The training set was further divided into training and validation sets. The validation of the dataset occurs simultaneously during training. Deep learning (DL) models were implemented using MATLAB 2023b owing to its better visualization and ease of use.

To obtain optimum results, the hyperparameters were adjusted. Epochs are the number of times the entire dataset is passed through the network for training. In this case, the dataset was trained for 30, 50, and 100 epochs, respectively. Initial learn rate is the amount of learning that happens at a step i.e. step size at which parameters are updated during training process. This was set to 0.001. The optimizer used was a Stochastic Gradient Descent with momentum (sgdm).

DL model performance was assessed using the specificity, sensitivity, Precision, NPV, Recall, F1 score.

## Results

In our study we included 100 symptomatic and asymptomatic cases.

The mean age and sex of the symptomatic and asymptomatic cases are shown in Table 1.

In our study, we analyzed ML models based on radiomic features and DL methods to predict LBP in symptomatic and asymptomatic cases.

**Feature reduction for ML model development:** The top 20 radiomic features for each lumbar vertebra and IVD were identified using a mutual information algorithm and are presented in Tables 3, 4.

**Table 3.** Showing the top 20 radiomic features selected at each vertebrae level for ML Models.

S.No.	Vertebrae	Features selected for ML models
**1**	**L1**	Dependence entropy, Run Length Non-Uniformity, Energy, Zone Entropy, Total Energy, Size Zone Non-Uniformity Normalized, Large Area Emphasis, Maximum 2D Diameter Row, Small Area Emphasis, Zone Variance, Robust Mean Absolute Deviation, Idn, Least Axis Length, Difference Variance, Strength, Imc2, Long Run High Grey Level Emphasis, Joint Entropy, entropy, Large Dependence High Grey Level Emphasis
**2**	**L2**	Surface Area, Dependence Entropy, Total Energy, Long Run High Gray Level Emphasis, Maximum 2D Diameter Row, Entropy, Idn, Imc2, Energy, Kurtosis, Least AxisLength, Small Dependence Low Gray Level Emphasis, Mean, Cluster Tendency, Run Percentage, Run Length Non Uniformity, Size Zone Non Uniformity Normalized, MCC, Long Run Low Gray Level Emphasis, Large Dependence Emphasis
**3**	**L3**	Least Axis Length, Idmn, Long Run High Grade Level Emphasis, Mean, Run Entropy, Run Length Non Uniformity Normalized, Strength, Kurtosis, Inverse Variance, Maximum3D Diameter, Run Variance, Sum Entropy, Auto Correlation, Maximum, Size Zone Non Uniformity Normalised, Short Run Emphasis, Skewness, Small Dependence High Gray Level Emphasis, Large Dependence High Gray Level Emphasis, Correlation
**4**	**L4**	Minor Axis Length, Large Dependence Emphasis, Gray Level Variance, Gray Level Non-Uniformity Normalized, Coarseness, Sum Squares, Difference Variance, Maximum 2D Diameter Row, Large Area High Gray Level Emphasis, Id, Elongation, Zone Entropy, Contrast, Maximum 2D Diameter Slice, Large Area Low Gray Level Emphasis, Mean, Large Dependence Low Gray Level Emphasis, Least Axis Length, Root Mean Squared, Low Gray Level Emphasis
**5**	**L5**	Contrast, Maximum, Small Area Low Gray Level Emphasis, Mean, Complexity, Least Axis Length, Range, Large Area Low Gray Level Emphasis, Idmn, Gray Level Non-Uniformity, Interquartile Range, Run Variance, 90 Percentile, Cluster Shade, Difference Entropy, Gray Level Variance, Maximum Probability, Gray Level Non Uniformity, Large Dependence Low Gray Level Emphasis, Run Entropy

**Table 4.** Showing the top 20 radiomic features selected at each IVD for ML Models.

S.No.	Intervertebral disc	Features selected for ML models
**1**	**L1-L2**	Sum Squares, Cluster Tendency, Zone Variance, Root Mean Squared, Short Run High Gray Level Emphasis, Joint Average, Sum Average, Large Area Emphasis, Gray Level Non-Uniformity Normalized, Entropy, Cluster Shade, Short Run Emphasis, High Gray Level Run Emphasis, Maximum 2D Diameter Row, Run Percentage, Dependence Entropy, Contrast, Median, Run Entropy, Interquartile Range
**2**	**L2-L3**	Maximum 2D Diameter Row, Short Run Emphasis, Maximum, High Gray Level Zone Emphasis, Maximum Probability, Difference Variance, Dependence Entropy, Id, Gray Level Non Uniformity Normalized, Contrast, Gray Level Non Uniformity Normalized, Gray Level Variance, High Gray Level Emphasis, Skewness, Range, Joint Entropy, Cluster Prominence, Run Variance, Low Gray Level Run Emphasis, Complexity
**3**	**L3-L4**	High Gray Level Zone Emphasis, Maximum 3D Diameter, Maximum, Range, Robust Mean Absolute Deviation, Auto correlation, Small Area High Gray Level Emphasis, Low Gray Level Zone Emphasis, Mean, Run Variance, Zone Entropy, Interquartile Range, Energy, Sum Entropy, Joint Average, Sum Average, Gray Level Variance, Long Run High Gray Level Emphasis, Cluster Prominence, Gray Level Normalized
**4**	**L4-L5**	Low Gray Level Emphasis, Dependence Entropy, Entropy, Minor Axis Length, Correlation, Small Area High Gray Level Emphasis, Maximum Probability, Difference Variance, Dependence Non Uniformity Normalized, Contrast, McC, Sum Average, Joint Average, Maximum 2D Diameter Column, Dependence Non Uniformity, Maximum 2D Diameter Row, Run Variance, Run Length Non Uniformity Normalized, Dependence Variance, Energy
**5**	**L5-S1**	Flatness, Zone Percentage, Least Axis Length, Entropy, Joint Entropy, Gray Level Non Uniformity Normalized, Joint Energy, Gray Level Non Uniformity, Size Zone Non Uniformity, Coarseness, Dependence Non Uniformity, Surface Area, Low Gray Level Zone Emphasis, Short Run High Gray Level Emphasis, Maximum, Cluster Shade, Short Run Emphasis, Dependence Non Uniformity Normalized, Range, Run Length Non Uniformity.

### Machine learning (ML) classifiers

In this study, ML methods such as Random Forest, Decision tree, Logistic regression, KNN, Adaboost were studied. The performance of the ML classifiers at the lumbar vertebrae and intervertebral disc using five-fold cross-validation is shown in Tables 5, 6 ^43^ for the five classifier models.

### Lumbar vertebrae

**Figure 5.**
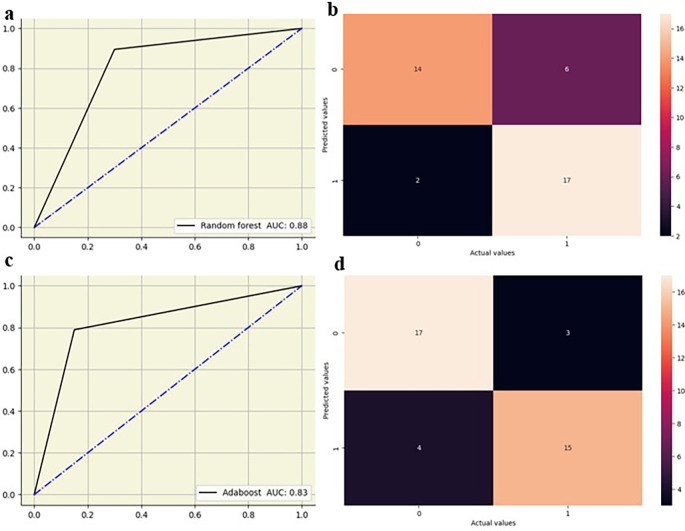
ROC curve and confusion matrix for random forest (a,b) and adaboost (c,d) at L4.

**Figure 6.**
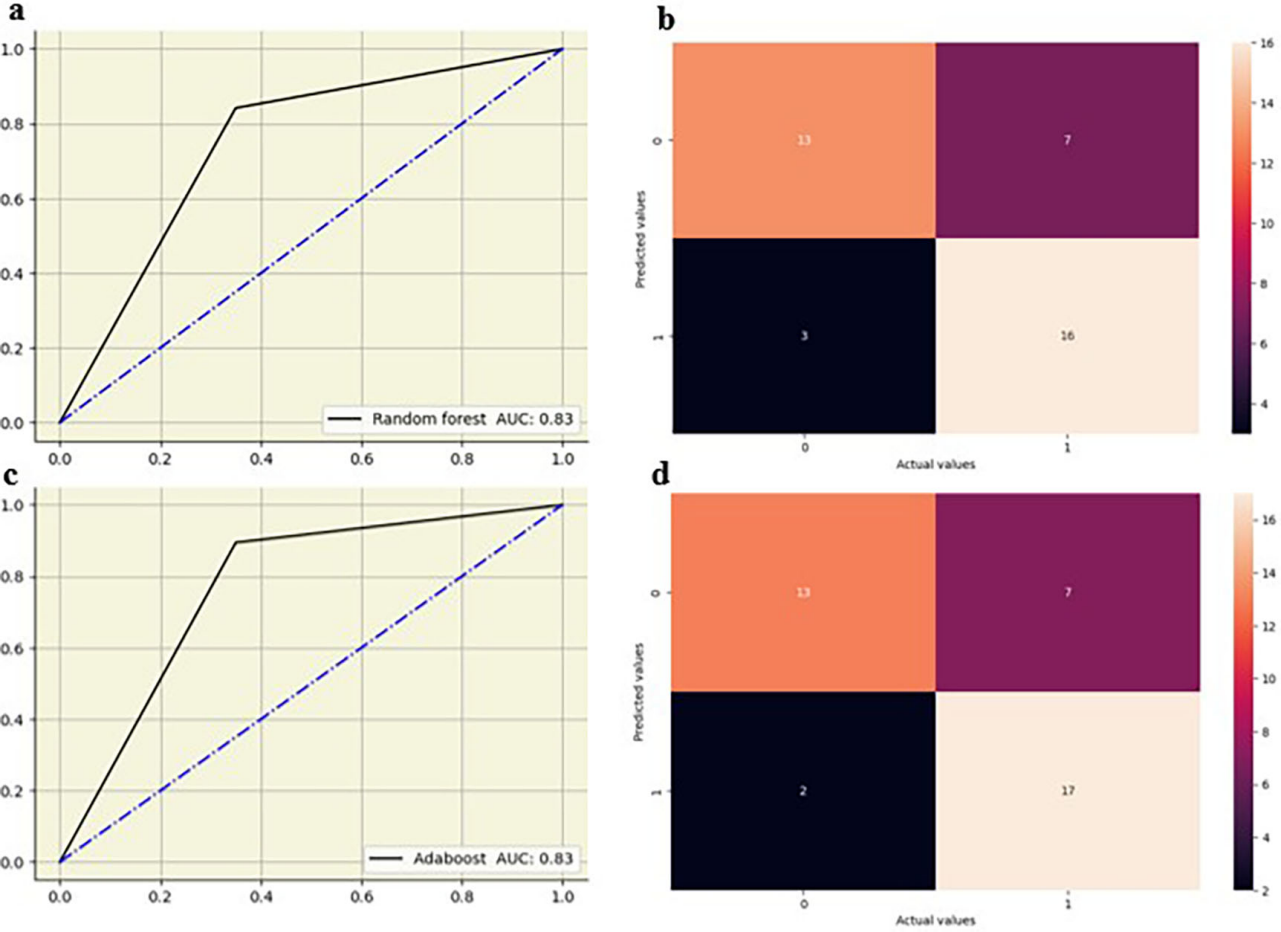
ROC curve and confusion matrix for random forest (a,b) and adaboost (c,d) at L5.

The random forest showed high performance across all lumbar vertebrae, with AUC values from 0.83 to 0.88 across all lumbar vertebrae. Decision tree models exhibited moderate performance with AUC values between 0.65 and 0.76, suggesting lower predictive accuracy compared to other models. Logistic regression performed well, particularly at L5 with an AUC of 0.82, and maintained good performance across other vertebral levels with AUC values from 0.73 0.79. KNN also showed strong performance, especially at L2-L4 vertebrae with AUC values of 0.79 to 0.83, and slightly lower AUC values at L1(0.70) and L5(0.68). AdaBoost demonstrated high performance at L2–L5 vertebrae with AUC values of 0.82 to 0.90, although its performance at L1 was moderate, with an AUC of 0.67. The ML models showed slightly improved performance at the lower vertebral levels (L4 and L5) compared to the upper vertebral levels (L1-L3) Figures 5, 6.

### Lumbar intervertebral disc

**Figure 7.**
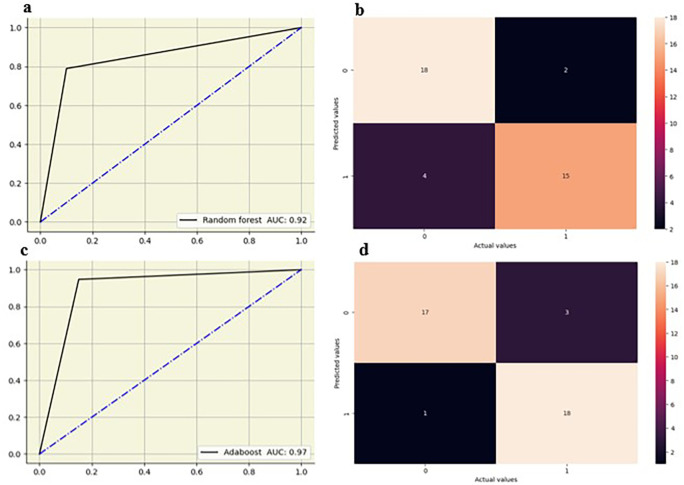
ROC curve and confusion matrix for random forest (a,b) and adaboost (c,d) at L5-S1 IVD.

Random forest showed strong performance at L2-L3, L3-L4 and L4-L5 IVD’s with AUCs of 0.88 and the highest at L5-S1 IVD (AUC-0.92). Decision tree models showed moderate performance, with the highest AUC at the L5-S1 IVD (0.85) and lower values at other disks, particularly at the L4-L5 IVD (AUC-0.65). Logistic regression showed the highest AUC at L3-L4 IVD (0.90) and maintained good performance at other disks, with AUC ranging from 0.79 0.87. KNN showed the highest AUC at the L4-L5 disk IVD (0.88) and moderately at other IVD disk between 0.73 and 0.78. Adaboost showed the highest AUC (0.97) at the L5-S1 IVD and exhibited strong results at the L2-L3 (0.86) and L3-L4 (0.83) IVD. The random forest and adaboost models showed high performance, particularly at the L5-S1 IVD ( Figure 7).

### Deep learning methods

The performance measures of the DL methods for LBP prediction are presented in Table 7.

**Table 7.** Showing the performance measures of DL methods for test dataset in prediction of LBP.

DL Model	Performance measures							
	Sensitivity	Specificity	Precision	NPV	FPR	Accuracy	F1 score	MCC
**ResNet50, 30 Epoch**	0.77	0.81	0.82	0.75	0.19	0.79	0.79	0.57
**GoogleNet, 30 Epoch**	0.85	0.86	0.86	0.85	0.14	0.85	0.86	0.71
**ResNet18, 30 Epoch**	0.80	0.89	0.91	0.77	0.10	0.84	0.85	0.69
**ResNet50, 50 epoch**	0.78	0.83	0.85	0.75	0.16	0.80	0.81	0.61
**GoogleNet, 50 Epoch**	0.83	0.85	0.85	0.83	0.15	0.84	0.84	0.68
**ResNet18, 50 Epoch**	0.79	0.91	0.92	0.75	0.09	0.84	0.85	0.69
**ResNet50, 100 Epoch**	0.78	0.83	0.85	0.75	0.16	0.80	0.81	0.61

NPV - Negative predictive value, FPR - False positive rate, MCC - Mathews correlation coefficient.

GoogleNet at 30 epochs outperformed the other DL models in terms of accuracy (0.85) and F1 score (0.86) for predicting LBP. ResNet 18 at 30 epochs had the second highest performance, with high accuracy (0.84) and F1 score (0.85). ResNet 50 showed consistent results at both 50 epochs (0.80-accuracy and 0.81-F1 score) and 100 epochs (accuracy-0.80 and F1 score-0.81), but with slightly lower performance metrics than GoogleNet and ResNet 18 at 30 epochs (accuracy-0.84 and F1 score-0.85).

## Discussion

In our study, we used ML and DL algorithms to predict LBP by using T2 weighted images of the lumbar spine. MRI studies have shown that a significant percentage of asymptomatic patients have abnormalities related to lumbar intervertebral discs. Imaging studies often fail to provide definitive answers regarding the source of pain. Imaging techniques are valuable tools for diagnosis and are often clinically inconclusive in identifying the precise etiology of low back pain. The high prevalence of asymptomatic abnormalities, risk of overdiagnosis, and lack of correlation between imaging findings and pain highlight the need for a cautious and judicious approach to the use of imaging in LBP management. Clinicians should rely on thorough clinical assessment and consider imaging findings as part of a broader diagnostic strategy rather than the sole determinant of patient care. ^34^^–^^36^

Our study noted that ML models showed improved performance at the lower vertebral levels (L4 and L5) compared to the upper vertebral levels (L1-L3) and random forest and adaboost models exhibited particularly high performance at the L5-S1 IVD. Abdollah et al. ^37^ reported that texture features extracted from T2 maps revealed significant textural differences in the L5-S1 lumbar IVD, upper and lower endplate regions, and the L4-5 lower endplate regions between individuals who are symptomatic and asymptomatic of LBP, which may not be apparent to the naked eye. The IVD and endplate zones of patients with LBP were more anisotropic, suggesting different patterns of degeneration due to varying patterns of collagen network destruction. Increased anisotropy may indicate fluid redistribution and changes in hydrostatic pressure, causing an uneven load distribution in pain-sensitive areas. Differences in Gray Level Co-occurrence Matrix features such as contrast, energy, and homogeneity provide additional evidence for the hypothesis of unique degeneration patterns in LBP. The random forest algorithm and Gini importance index indicate energy as a unique feature for classification. Ketola et al. ^38^ also reported difference in T2 weighted images analyzed using logistic regression to classify textural features based on a pain questionnaire in a sample of 518 subjects. The best classification accuracy (83%) and AUC (0.91) were achieved at the lowest two IVDS, with a specificity score of 83% and a sensitivity score of 82%. These results suggest that texture features in the lower lumbar discs (L4-L5 and L5-S1) are more predictive of LBP, supported by the findings of increased anisotropy and genetic correlations. Another study by Aggarwal et al. ^39^ reported that decreased L2 and L4 disc heights significantly predicted LBP. They also reported that thickening of the ligamentum flavum, particularly at the lower lumbar levels, contributes to spinal stenosis and LBP.

The DL models used in our study were useful for predicting LBP using MRI. GoogleNet with 30 epochs showed the highest performance with an accuracy of 0.85 and an F1 score (0.86) for predicting LBP. Won et al. ^40^ employed a CNN to automatically grade spinal stenosis on MRI images of 542 patients, obtaining accuracy measures of 83.0% and 77.9% in comparison to the ground truth assessed by two separate doctors. Jamaludin et al. ^41^^,^^42^ developed a CNN that segments the vertebrae and intervertebral discs with an accuracy of 95.6%. This model also identifies disc narrowing, marrow changes, endplate defects, spondylolisthesis, and central canal stenosis, and performs Pfirrmann grading with accuracy percentages ranging from 70.1% and 95.4%. Additionally, it can directly highlight abnormalities of the IVD and vertebrae using heatmaps, referred to as evidence hotspots ^41^^,^^42^

Our study had a few limitations. First, the sample size is sufficient for the initial analysis; a larger sample size could provide more robust results and improve the reliability of machine learning (ML), and deep learning (DL) models. Second, manual segmentation of the lumbar vertebrae and IVD is time-consuming and subject to inter-operator variability. Automated segmentation methods can enhance reproducibility and efficiency. Third, the study did not include risk factors, radiological findings, or their role in assessing LBP using machine learning methods.

## Conclusion

Our study found that ML classifiers, such as random forest and adaboost, exhibited the highest performance, particularly in the lower lumbar vertebrae and IVD, while decision tree and logistic regression models showed moderate performance in the prediction of LBP. For DL methods, GoogleNet achieved the best results at 30 epochs, followed closely by ResNet, which demonstrated high precision and specificity. Our findings highlight the potential of advanced ML and DL techniques for accurately predicting LBP, with random forest, AdaBoost, and GoogleNet showing the most promising results.

## Ethical approval

This was a prospective, case-control study. The institutional ethical committee (IEC2:179/2023) was obtained from Kasturba medical college and Hospital, Manipal, India on 20 ^th^ July 2023, followed by the Clinical trial registry (CTRI) registration: CTRI/2023/08/056954, 25/08/2023, https://ctri.nic.in/Clinicaltrials/login.php. Written Informed consent (IC) was obtained from all the participants.

## Data Availability

Figshare: F1000 ML and DL Data, https://doi.org/10.6084/m9.figshare.26394847.v2. ^43^ This project contains following underlying data:
• Radiomic features of lumbar spine cases (demographic characteristics of cases, radiomic features, spreadsheet) • Radiomic features of controls of the lumbar spine (demographic characteristics of controls, radiomic features–spreadsheet) • Anonymous Images cases (MRI JPEG images) • Anonymous Images controls (MRI JPEG images) Data are available under the terms of the Creative Commons Attribution 4.0 International license (CC-BY 4.0). Figshare: F1000 ML and DL Data, https://doi.org/10.6084/m9.figshare.26394847.v2 ^43^ This project contains following Extended data:
•
Table 5 and 6 • Supplementary file 1
