# Supplementary material for: Role of Artificial intelligence model in prediction of low back pain using T2 weighted MRI of Lumbar spine
Source: F1000Res. 2024 Oct 10;13:1035. Originally published 2024 Sep 10. [Version 2] doi: 10.12688/f1000research.154680.2 (PMC11525099; doi:10.12688/f1000research.154680.2)
Supplement: Peer review history

# Reviewer report: Reviewer response for version 2

**DOI:** 10.5256/f1000research.173049.r335609

**Published:** 2024-11-18

**Authors:** Eugene Ozhinsky

**Affiliations:** 1 University of California San Francisco, San Francisco, California, USA

The manuscript describes a study using machine learning and deep learning techniques to predic whether the patients had lower-back pain. The study addresses an important issue of diagnosing lower-back pain. Here is a list of questions the authors could use to improve the manuscript:

 Introduction:

 Why is it useful to predict LBP from MR images? How can it help the patients?

 Please cite previous work using ML and DL to study LBP. How is your study different?

 Methods:

 I don't think this study is a prospective case-control study. The data was analyzed after it was collected and the patients were not followed up on.

 How were the patients recruited? What conditions did the controls have?

 Which software was used to extract radiomic features?  

 Please clarify if there was a separate test set not used for finetuning.

 The manuscript states that "The input data were split into five equal parts: four groups for training and five for testing...". It is not clear how 4 and 5 groups were generated from 5 equal parts.

 How many MRI slices were used from each subject? How were the slices chosen? How were the features from these slices combined? How did DL models work with the slices if there were more than one slice?

 Discussion:

 How did your study improve upon previous approaches in literature?

Is the work clearly and accurately presented and does it cite the current literature?

Partly

If applicable, is the statistical analysis and its interpretation appropriate?

Yes

Are all the source data underlying the results available to ensure full reproducibility?

Yes

Is the study design appropriate and is the work technically sound?

No

Are the conclusions drawn adequately supported by the results?

Yes

Are sufficient details of methods and analysis provided to allow replication by others?

Partly

Reviewer Expertise:

Radiology, Magnetic Resonance Imaging, Machine Learning, Musculoskeletal Imaging, Focused Ultrasound

I confirm that I have read this submission and believe that I have an appropriate level of expertise to state that I do not consider it to be of an acceptable scientific standard, for reasons outlined above.

# Reviewer report: Reviewer response for version 2

**DOI:** 10.5256/f1000research.173049.r335604

**Published:** 2024-10-30

**Authors:** Shashi Kumar Shetty

**Affiliations:** 1 K S Hegde Medical Academy, Mangalore,, Karnataka, India

**Major comments:**

- The study effectively explores the role of machine learning (ML) and deep learning (DL) models in predicting low back pain using T2 weighted MRI images. This novel method has substantial potential for improving non-invasive diagnostic accuracy in LBP.
- The use of mutual information for radiomic feature selection and reduction at each lumbar vertebral body and disc space has been clearly highlighted in the study which would serve as reference for future studies.
- The choice of Machine learning (ML) models such as random forest and ad boost, allows study to benefit from models known for their robustness and interpretability, which is especially important in medical diagnostics.
- The inclusion of convolution neural networks (CNN) such as GoogleNet and ResNet  (transfer learning) methods reflects advanced approach, leveraging the strength of these models to capture medical images.
- The use of balanced datasets in each class (symptomatic and asymptomatic cases) in binary classification tasks is clearly understandable from the manuscript.

**Minor comments:**

- For quick understanding of readers, raw MRI images of lumbar spine were used for deep learning and radiomics were utilized for deep learning may be mentioned in discussion.

The article is well-structured, informative, and presents a promising approach to making use of AI for non-invasive diagnosis, and improved patient outcomes in musculoskeletal health.

Is the work clearly and accurately presented and does it cite the current literature?

Yes

If applicable, is the statistical analysis and its interpretation appropriate?

Yes

Are all the source data underlying the results available to ensure full reproducibility?

Yes

Is the study design appropriate and is the work technically sound?

Yes

Are the conclusions drawn adequately supported by the results?

Yes

Are sufficient details of methods and analysis provided to allow replication by others?

Yes

Reviewer Expertise:

Radiology, artificial intelligence, radiation protection

I confirm that I have read this submission and believe that I have an appropriate level of expertise to confirm that it is of an acceptable scientific standard.

# Reviewer report: Reviewer response for version 2

**DOI:** 10.5256/f1000research.173049.r335830

**Published:** 2024-10-30

**Authors:** Chandrasekhar Priyanka

**Affiliations:** 1 Sri Ramachandra Institute of Higher Education and Research (Deemed to be University), Chennai, Tamil Nadu, India; 2 Department of Allied Health Sciences, The Apollo University, Chittoor, Andhra Pradesh, India

**Major comments:**

- The study represents a progressive approach to diagnose low back pain (LBP), utilizing radiomics based machine learning (ML) and T2 weighted MRI image based deep learning (DL) models. In musculoskeletal imaging, precise pain source identification is crucial but frequently difficult with conventional MRI imaging, in such cases, Artificial intelligence plays a major role for non-invasive diagnosis.
- The use of Delphi definitions of low back pain (DOLBaPP) questionnaire is excellent. It provides a standardized way to assess LBP prevalence, helping ensure consistency in identifying symptomatic and asymptomatic patients. The 12-month criterion for identifying cases is well chosen, as it allows for identification of more chronic presentations of LBP. Similarly, the clear mentioning of controls avoids ambiguity, ensuring clear differentiation between cases and controls.
- Balanced datasets with equal representation of symptomatic and asymptomatic groups were significant strength, as it reduces class balance issues that often skew artificial intelligence models performance in medical studies.
- The article explained the rationale for the selection of various ML and DL models, and the strengths of each algorithm in the context of LBP prediction.

**Minor Comments:**

- Typographical errors like F1 score formula can be corrected.

The study contributes to the growing field of AI in medical imaging and health care, emphasizing the importance of integrating AI tools into clinical workflows for better management of LBP.

Is the work clearly and accurately presented and does it cite the current literature?

Yes

If applicable, is the statistical analysis and its interpretation appropriate?

I cannot comment. A qualified statistician is required.

Are all the source data underlying the results available to ensure full reproducibility?

Yes

Is the study design appropriate and is the work technically sound?

Yes

Are the conclusions drawn adequately supported by the results?

Yes

Are sufficient details of methods and analysis provided to allow replication by others?

Yes

Reviewer Expertise:

Magnetic Resonance Imaging, Artificial intelligence in health care

I confirm that I have read this submission and believe that I have an appropriate level of expertise to confirm that it is of an acceptable scientific standard.

# Reviewer report: Reviewer response for version 2

**DOI:** 10.5256/f1000research.173049.r330660

**Published:** 2024-10-12

**Authors:** Tarun Gangil

**Affiliations:** 1 The institute of cancer research, London, UK

**Question 1: Authors should highlight , why they have chosen a wide range of machine learning algorithms, clustering algorithm (KNN) and deep learning algorithms such as ResNet and GoogleNet ?**

 No concerns anymore.

**Question 2:** **Do the analysis were performed only on the radiomics dataset ? If that is the case then why do they have used segmentation algorithms such as ResNet and GoogleNet ?**

 If I understand the author's response correctly, the MRI images were used directly, and the analysis performed was a classification task, not a segmentation task. Also, could it be mentioned explicitly, if not otherwise stated in the manuscript, that if it is a binary classification task, then how many samples for each class have the authors used for the analysis? 

**Question 2:** **Do the analysis were performed only on the radiomics dataset ? If that is the case then why do they have used segmentation algorithms such as ResNet and GoogleNet ?**

 No concerns anymore

**Question 3:** **If MRI images are used in analysis, without radiomics dataset, then what is the average sensitivity of the ground truth segmentation mask across all samples?**

 No concerns anymore

**Question 4: Do authors have used Images as an input in the analysis, apart from the radiomics dataset, directly in the AI models.**

 No concerns anymore

**Question 5:** **Also, highlight about class variability, given it is a binary classification problem or Multiclass?**

 Please mention about the number of samples from each class and also I believe the formula of F1 score needs correction. If it is convenient then use the equations settings from word to write any equations.

**Question 6:** **Also, post analysis , it is needed for the authors to highlight the clinical significance of the results obtained from the classification algorithms.**

 No concerns.

 Question 7: Can authors highlight the features which are having high importance towards the classification? I can suggest the use of SHAP analysis will be appropriate in this case, and further, the variables highlighted as important by the best-performing ML model can be compared with the clinical literature to conclude the findings of this research.

Is the work clearly and accurately presented and does it cite the current literature?

Partly

If applicable, is the statistical analysis and its interpretation appropriate?

No

Are all the source data underlying the results available to ensure full reproducibility?

Yes

Is the study design appropriate and is the work technically sound?

Partly

Are the conclusions drawn adequately supported by the results?

No

Are sufficient details of methods and analysis provided to allow replication by others?

Partly

Reviewer Expertise:

Artificial Intelligence in Oncology, Image Processing , Deep Learning and Machine Learning

I confirm that I have read this submission and believe that I have an appropriate level of expertise to confirm that it is of an acceptable scientific standard, however I have significant reservations, as outlined above.

# Reviewer report: Reviewer response for version 1

**DOI:** 10.5256/f1000research.169735.r325460

**Published:** 2024-10-01

**Authors:** Tarun Gangil

**Affiliations:** 1 The institute of cancer research, London, UK

Authors should highlight , why they have chosen a wide range of machine learning algorithms, clustering algorithm (KNN) and deep learning algorithms such as ResNet and GoogleNet ?

 Do the analysis were performed only on the radiomics dataset ? If that is the case then why do they have used segmentation algorithms such as ResNet and GoogleNet ?

 If MRI images are used in analysis, without radiomics dataset, then what is the average sensitivity of the ground truth segmentation mask across all samples?

 Do authors have used Images as an input in the analysis , apart from the radiomics dataset, directly in the AI models.

 Also, highlight about class variability, given it is a binary classification problem or Multiclass ? 

 Also, post analysis , it is needed for the authors to highlight the clinical significance of the results obtained from the classification algorithms.

Is the work clearly and accurately presented and does it cite the current literature?

Partly

If applicable, is the statistical analysis and its interpretation appropriate?

No

Are all the source data underlying the results available to ensure full reproducibility?

Yes

Is the study design appropriate and is the work technically sound?

Partly

Are the conclusions drawn adequately supported by the results?

No

Are sufficient details of methods and analysis provided to allow replication by others?

Partly

Reviewer Expertise:

Artificial Intelligence in Oncology, Image Processing , Deep Learning and Machine Learning

I confirm that I have read this submission and believe that I have an appropriate level of expertise to confirm that it is of an acceptable scientific standard, however I have significant reservations, as outlined above.

# Response

**Published:** 2024-10-05

**Authors:** Saikiran Pendem

**Affiliations:** Medical Imaging Technology, Manipal Academy of Higher Education, Manipal, Karnataka, India

**Question 1: Authors should highlight , why they have chosen a wide range of machine learning algorithms, clustering algorithm (KNN) and deep learning algorithms such as ResNet and GoogleNet ?**

**Ans: **The advantages for using wide range of machine learning algorithms, cluster algorithm (KNN) and deep learning algorithms such as ResNet and GoogleNet were provided in the methodology.

 We have utilized a wide range of ML classifiers since different classifiers may perform better with data features and this allows in through benchmarking and the selection of an optimal model for a specific problem. Each ML method has its own advantages, random forest excels in robust and accuracy, decision tree offers interpretability, logistic regression is effective for linear relationships, KNN is good for smaller dataset, adaboost improves performance by merging weak learners.

 GoogleNet and ResNet were chosen due to their powerful ability to learn complex patterns in data, especially in image analysis, medical diagnostics and classification problems. Both are exceptional at automatically learning deep features particularly involving images, where they can capture complex and minute details. GoogleNet inception modules process input using parallel convolution layers with varying kernel sizes, ehancing efficiency by capturing features at different scales with fewer parameters. ResNet solves the vanishing gradient issue, making it possible to train very deep networks efficiently. This permit learning more complicated representations improves performance and tasks like image classification and object recognition.

**Question 2:** **Do the analysis were performed only on the radiomics dataset ? If that is the case then why do they have used segmentation algorithms such as ResNet and GoogleNet ?**

**Ans: **The radiomics features were extracted for the purpose of using the ML classifiers. As for the Deep Learning models, there were no radiomics features. These models are black boxes which extract the features from the images without human intervention and classify them.

**Question 3:** **If MRI images are used in analysis, without radiomics dataset, then what is the average sensitivity of the ground truth segmentation mask across all samples?**

**Ans: **In case of Deep Learning method, the MRI images are used for the analysis (classification) without radiomic features. Hence, no ground truth masks. The classification model gives sensitivity between the range of 75-85%.

**Question 4: Do authors have used Images as an input in the analysis, apart from the radiomics dataset, directly in the AI models.**

**Ans: **Yes, for the deep learning models, direct images for the classification task.

**Question 5:** **Also, highlight about class variability, given it is a binary classification problem or Multiclass?**

**Ans: **It is a binary classification problem which helps in predicting the LBP. Class variability impacts evaluation metrics such as sensitivity, specificity, precision, and F1-score. The more variability there is within and between classes, the more robust the model needs to be.

 The same is included in the methodology section.

**Question 6:** **Also, post analysis , it is needed for the authors to highlight the clinical significance of the results obtained from the classification algorithms.**

**Ans: **The clinical significance of the results obtained from the classification algorithms were included in the discussion section.

 According to our study, ML and DL models could provide more efficient, reliable, noninvasive diagnostic insights by accurately identifying abnormalities in the lumbar vertebrae and intervertebral discs (IVDs), even in cases where conventional MRI image assessments were inconclusive. By improving the ability to predict LBP, ML and DL algorithms could guide better clinical decision making, reduce unnecessary surgical interventions.

# Reviewer report: Reviewer response for version 1

**DOI:** 10.5256/f1000research.169735.r325453

**Published:** 2024-09-24

**Authors:** Chandrasekhar Priyanka

**Affiliations:** 1 Sri Ramachandra Institute of Higher Education and Research (Deemed to be University), Chennai, Tamil Nadu, India; 2 Department of Allied Health Sciences, The Apollo University, Chittoor, Andhra Pradesh, India

**Major comments:**

 The research article presents a case-control study to assess machine and deep learning models for prediction of low back pain using T2-weighted MRI images of lumbar spine.

 The utility of mutual information for radiomic feature selection is a good approach; however, the reasons for selecting this over frequently used feature selection method such as least absolute shrinkage and selection operator (LASSO) is not adequately justified.

 Though the study employed five-fold cross validation, it would benefit from discussing additional methods such as bootstrapping or using different validation splits.

 The study briefly mentions hyperparameter tuning, but it did not discuss in detail whether any steps were taken to reduce overfitting.

 Future research directions in how AI model predictions should be clinically interpreted and implications of incorporating these AI models into the diagnostic workflow can be provided.

**Minor comments:**

 Inconsistent capitalization to be corrected – the words “random forest” and “adaboost” are capitalized inconsistently.

 The article effectively demonstrates the use of machine and deep learning models in accurately predicting low back pain and provides promising contributions to knowledge about the use of AI in healthcare.

Is the work clearly and accurately presented and does it cite the current literature?

Yes

If applicable, is the statistical analysis and its interpretation appropriate?

I cannot comment. A qualified statistician is required.

Are all the source data underlying the results available to ensure full reproducibility?

Yes

Is the study design appropriate and is the work technically sound?

Yes

Are the conclusions drawn adequately supported by the results?

Yes

Are sufficient details of methods and analysis provided to allow replication by others?

Yes

Reviewer Expertise:

Magnetic Resonance Imaging, Artificial intelligence in health care

I confirm that I have read this submission and believe that I have an appropriate level of expertise to confirm that it is of an acceptable scientific standard.
